# Comparison of Maternal Serum Neuropilin-1 (NRP-1) and Fetal Cord Blood NRP-1 Concentrations in Between Normotensive Pregnant Women and Those with Preeclampsia

**DOI:** 10.3390/jcm14113718

**Published:** 2025-05-26

**Authors:** Simge Tosun, Raziye Torun, Mehmet Ferdi Kinci, Saliha Aksun, Mustafa Sengul

**Affiliations:** 1 Department of Obstetrics and Gynecology, Cukurca State Hospital, 30700 Hakkari, Turkey; simge.tosun@hotmail.com; 2 Department of Perinatology, Izmir City Hospital, 35510 Izmir, Turkey; 3 Department of Obstetrics and Gynecology, Izmir City Hospital, 35510 Izmir, Turkey; drferdikinci@gmail.com; 4 Department of Medical Biochemistry, Faculty of Medicine, Izmir Katip Celebi University, 35620 Izmir, Turkey; saliha.aksun@ikc.edu.tr; 5 Department of Obstetrics and Gynecology, Faculty of Medicine, Izmir Katip Celebi University, 35620 Izmir, Turkey; dr.mustafasengul@gmail.com

**Keywords:** hypertension, pregnancy, neuropilin-1, preeclampsia

## Abstract

**Background:** The aim of this study was to compare the levels of Neuropilin-1 (NRP-1) in maternal plasma and fetal cord blood plasma between pregnancies complicated by preeclampsia (PE) and those in normotensive pregnant women. **Materials and Methods:** This prospectively designed study included 53 pregnant women aged 18 years or older and at least 20 weeks into gestation, who were admitted to the Maternity Department of Izmir Katip Çelebi University Atatürk Training and Research Hospital. The patient group consisted of 28 pregnant women who met the diagnostic criteria for PE, while the control group included 25 normotensive pregnant women. The diagnosis of PE was established based on the 2020 diagnostic criteria of the American College of Obstetricians and Gynecologists (ACOG). After detailed anamnesis, blood samples were collected immediately after delivery in EDTA tubes to assess serum NRP-1 levels. These samples included maternal blood, fetal cord blood, and additional tests such as CBC, liver and kidney function tests, serum electrolytes, spot urinalysis, prothrombin time (PT), and activated partial thromboplastin time (APTT). **Results:** There was a statistically significant difference between the two groups in terms of gestational week, presence of comorbidities, hypertension (HT), diabetes mellitus (DM), history of PE, and protein detected in spot urine examinations. Pregnant women in the PE group had significantly higher rates of comorbidities, HT, and DM compared to the control group (*p* < 0.001, 0.002, and 0.007, respectively). No statistically significant differences were observed between the two groups regarding hemoglobin, platelet count, aspartate aminotransferase (AST), alanine aminotransferase (ALT), or fetal cord plasma NRP-1 levels (*p*: 0.736, 0.831, 0.561, and 0.734, respectively). However, a statistically significant difference was found in maternal plasma NRP-1 levels (*p*: 0.02), which were lower in the control group compared to the PE group (median: 473.3 pg/mL vs. 587.7 pg/mL, respectively). The optimal cut-off value for maternal plasma NRP-1 to predict PE, with the best sensitivity and specificity, was determined to be 358.4 pg/mL. Among the study participants, 40 pregnant women had maternal plasma NRP-1 levels above the cut-off value, while 13 had levels below it. PE occurred significantly more frequently in the high NRP-1 group than in the low group. When demographic and clinical characteristics were analyzed, a statistically significant but weak positive correlation was found between body mass index (BMI) and maternal plasma NRP-1 levels (*p*: 0.02, Rho: 0.304). No strong or statistically significant relationships were identified with other variables. There was no significant difference in fetal cord plasma NRP-1 levels between the PE group and the normotensive group. In contrast, maternal plasma NRP-1 levels were significantly higher in the PE group. The cut-off value for maternal plasma NRP-1, providing optimal sensitivity and specificity for predicting PE, remained 358.4 pg/mL. **Conclusions:** While further studies involving larger cohorts of pregnant women from diverse racial backgrounds and various hospitals are needed to better understand the relationship between NRP-1 and PE, maternal NRP-1 concentration shows promise as a diagnostic marker.

## 1. Introduction

Preeclampsia (PE) is a leading cause of maternal and perinatal morbidity and mortality, occurring after 20 weeks of gestation and characterized by new-onset hypertension accompanied by proteinuria, maternal end-organ damage, or uteroplacental dysfunction (e.g., intrauterine growth restriction [IUGR] or angiogenic imbalance) [1]. Each year, an estimated 4 million cases of PE are reported worldwide, resulting in the deaths of more than 70,000 women and 500,000 newborns [1,2].Previously, proteinuria was a required criterion for diagnosis. However, the American College of Obstetricians and Gynecologists (ACOG) revised its diagnostic criteria for PE in 2013. The updated criteria include a systolic blood pressure of ≥140 mmHg or a diastolic blood pressure of ≥90 mmHg, along with any of the following: proteinuria, thrombocytopenia, renal failure, liver dysfunction, pulmonary edema, or cerebral and visual disturbances [3].

Women who develop PE during pregnancy face an increased risk of cardiovascular diseases, stroke, chronic heart failure, and diabetes later in life [4]. For babies born from pregnancies complicated by PE, adverse outcomes such as preterm delivery, perinatal death, neurodevelopmental delays, and an elevated risk of cardiovascular and metabolic diseases in adulthood have been observed [1]. Consequently, PE imposes a significant lifelong economic burden on society, stemming from both maternal and fetal morbidity [5]. PE is classified based on gestational age. According to the International Society for the Study of Hypertension in Pregnancy (ISSHP), PE is categorized as preterm (<37 weeks of gestation), term (≥37 weeks of gestation), or postpartum. For research purposes, PE is often further divided into early-onset (<34 weeks of gestation) and late-onset (≥34 weeks of gestation) subtypes. Although these classifications are not routinely used in clinical practice, they are employed to analyze study populations and reflect differences in etiology [6]. Although the mechanism of PE has not been fully elucidated, maternal, fetal, and paternal factors are known to contribute to its etiology [7]. The pathogenesis of PE involves a mismatch between maternal tissue and the placenta, primarily due to inadequate trophoblast invasion and insufficient remodeling of the myometrial spiral arteries. This results in a disrupted balance between pro-angiogenic and anti-angiogenic proteins in the maternal circulation, driven by a hypoxic–ischemic microenvironment [3]. In PE, placentation is hindered by elevated levels of anti-angiogenic factors, such as soluble FMS-like tyrosine kinase-1 (sFlt-1), which inhibits the binding of Vascular Endothelial Growth Factor (VEGF) and placental growth factor (PIGF) to VEGFR-2 in endothelial cells. Additionally, soluble endoglin (sEng) is released, which disrupts TGF-β signaling and reduces the bioavailability of endothelial nitric oxide synthase (eNOS), a key vasodilator [8]. The hypoxic–ischemic placental environment further exacerbates the condition by promoting the release of pro-inflammatory cytokines, including Tumor Necrosis Factor-alpha (TNF-α) and interleukins (IL-1, IL-6, IL-8), as well as syncytiotrophoblast microparticles (STBMs). These factors inhibit the production of eNOS and prostaglandin I2, both essential for effective vasodilation [8]. NRP-1 is a transmembrane protein with high affinity for VEGF165 and PIGF. VEGF-A, a key mediator of angiogenesis, promotes endothelial cell migration and proliferation in the presence of NRP-1, despite its weak affinity for VEGFR-2. NRP-1 binds to VEGFR-2 in the presence of VEGF165, activating protein kinase B and stimulating phosphatidylinositol-3 kinase (PI3K), thereby facilitating angiogenesis. Beyond its angiogenic role, NRP-1 supports peripheral tolerance by promoting interactions between dendritic cells and regulatory T (Treg) cells, which are critical for immune modulation. These pro-angiogenic and immune functions make NRP-1 indispensable for a successful pregnancy [9]. NRP-1 plays a vital role in establishing and maintaining the maternal–fetal microenvironment. It is expressed in the decidua, syncytiotrophoblast, extravillous trophoblast cells, placental villous vessels, and vascular endothelial cells throughout pregnancy, with the highest expression observed in the first trimester [10]. The NRP-1 molecule, which plays an important role in the initiation of angiogenesis, plays a role in tip cell formation [11]. A subset of a vessel’s dormant endothelial cells lose their pericyte sheath in response to angiogenic signaling. It becomes ready to migrate by adhering to adjacent cells and less to the basement membrane. Migration is directed by the tip cell, a modified endothelial cell in the presence of molecules associated with angiogenesis. When a tip cell is formed, adjacent endothelial cells preferentially differentiate into stalk cells that proliferate to form vessels. VEGF, VEGFR-2, and NRP-1 promote stem cell formation, whereas NOTCH ligands DLL4 and JAG-GED1 inhibit it. The NOTCH ligands DLL4 and JAG-GED1 activate stem cells. The balance between these ensures the orientation of stalk cells and tip cells. Stalks develop lumen under the influence of vascular endothelium–cadherin, VEGF and other molecules. It forms vascular sprouts. Although angiogenesis steps have not been demonstrated in human fetal–placental vascular bed formation, it is known that NRP-1 is involved in decidual vascular development in a mouse model and in humans during early gestation [12]. The NRP-1 gene specifically binds VEGF165 and increases VEGF165 binding to VEGFR2 in vascular endothelial and tumor cells [13,14]. Angiogenesis is triggered by hypoxia in humans, which increases NRP-1 and VEGF production [15]. Experiments on mice have shown that NRP-1 and VEGF support angiogenesis by increasing during the implantation process. In these studies, VEGF164 is the predominant isoform of VEGF in the first 8 days of implantation. NRP-1 and VEGFR2 proliferate in the decidual bed [12].

This elevated expression during early pregnancy supports new vessel formation, facilitating implantation. NRP-1 produced by Treg cells also contributes to Treg cell activity and long-term suppression of CD8+ cells. Furthermore, NRP-1 is essential for healthy fetal growth.

Given these mechanisms, the tight regulation of NRP-1 expression during pregnancy is crucial [7]. Studies have shown that NRP-1 expression is decreased in pregnant women with PE. However, another study using a mouse model demonstrated increased NRP-1 expression in the placental villous layer of PE pregnancies. Additionally, RNA-binding protein quaking I-5 (QKI-5) and NRP-1 expressions were found to be reduced in the placenta and trophoblasts under hypoxic conditions associated with PE [16]. These findings suggest that NRP-1 plays a significant role in the development and progression of PE.

In this study, we aimed to investigate the role of NRP-1 in the pathogenesis of PE by comparing maternal serum and fetal cord blood plasma NRP-1 levels between pregnancies complicated by PE and those in the control group.

## 2. Materials and Methods

Human NRP 1 microeliza kit Elabscience (Human NRP1, neuropilin 1, catalog no: E-EL-H6164, Houton, TX, USA) was used in the study.

This prospective study included 53 pregnant women aged over 18 years and with a gestational age of more than 20 weeks, who were admitted to the Labor and Delivery Department of Izmir Katip Çelebi University Atatürk Training and Research Hospital. The patient group consisted of 28 pregnant women who met the diagnostic criteria for PE, while the control group comprised 25 normotensive pregnant women. The study was initiated following approval from the Izmir Katip Çelebi University Atatürk Training and Research Hospital Clinical Research Ethics Committee, dated 8 May 2018, and numbered 2011-KAEK-252018/05-02. Pregnant women under 18 years of age, those with a gestational age of less than 20 weeks, and those with history of hypertension in previous pregnancies were excluded from the study. Participants who met the inclusion criteria were informed about the study and were enrolled after providing their informed consent.

The diagnosis of PE was based on the ACOG 2020 diagnostic criteria for PE. These criteria are previously normotensive and blood pressure values measured at rest after the 20th week of gestation at least 4 h apart with systolic ≥ 140 mmHg and diastolic ≥ 90 mmHg or systolic blood pressure ≥ 160 mmHg or diastolic blood pressure ≥ 110 mmHg, proteinuria 1+ in a strip urine sample (if quantitative measurement is not possible), protein (mg/dL)/creatinine (mg/dL) ratio ≥ 0.3 mg/mg or proteinuria ≥ 0.3 g in 24 h urine, or thrombocytopenia (<100,000/mm^3^) for the diagnosis of PE in patients with hypertension after 20 weeks of gestation but without proteinuria, at least 2-fold elevation of normal transaminase levels in the liver, serum creatinine > 1.1 mg/dL (97.2 micromol/L) or doubling of creatinine concentration without other renal pathology, cerebral or visual symptoms, and pulmonary edema.

After each pregnant woman rested for 5 min, blood pressure measurements were taken using a calibrated sphygmomanometer with an appropriately sized cuff positioned at heart level. These measurements were performed by trained healthcare personnel in accordance with the 2013 guidelines for the management of arterial hypertension, published jointly by the European Society of Hypertension (ESH) and the European Society of Cardiology (ESC). If the systolic or diastolic blood pressure differed by more than 5 mmHg in at least two of the measurements, additional readings were taken. Each repetition was performed after an adequate rest interval.

After recording the medical history of pregnant women who were admitted to the delivery room and agreed to participate in the study, routine tests—including CBC, liver and kidney function tests, serum electrolytes, spot urinalysis, prothrombin time (PT), and activated partial thromboplastin time (APTT)—were performed. Maternal blood and fetal cord blood were collected in EDTA tubes immediately after delivery for the comparison of serum NRP-1 levels. The samples were then centrifuged at 5000 RPM for 5 min. After centrifugation, the plasma was separated and stored at −80 °C until analysis. To minimize variability between tests, samples were analyzed in batches. Hemolyzed plasma was excluded from the study, and NRP-1 levels were measured using the enzyme-linked immunosorbent assay (ELISA) method. The standard HRP1 concentration included in the kit is 1000 pg/mL.

The human NRP 1 microeliza kit Elabscience (Human NRP1, neuropilin 1, catalog no: E-EL-H6164, Houston, Teksas, USA) was used in the study. The standard HRP1 concentration in the kit is 1000 pg/mL. The microeliza test principle and steps of the study procedure are as follows:

1. The microeliza method works using the sandwich ELISA principle. In this method, 100 microliters each of the main standard at a concentration of 1000.00 pg/mL and standard samples of different concentrations prepared by dilution from this standard and serum samples of the study were added to 96-well plates coated with monoclonal antibodies against human sNRP-1, one sample per well. The reaction wells on the 96-well test plate were identified and recorded for each sample so that the standard and study samples seeded in the wells could be labeled. Standards and working samples at all concentrations used were inoculated twice each to ensure a replicate study.
2. After seeding of samples and standards, Elisa plates were incubated at 37 °C for 90 min for the binding of sNRP-1 ligands in samples and standards with antibodies against sNRP-1, which were ready coated in the wells.
3. At the end of the incubation period, the liquid material in the Elisa plate was drained and the biotinylated antibody against human sNRP-1 in the test kit was added to 100 microliters per well. The Elisa plate was incubated at 37 °C for 60 min to allow the biotinylated antibody to bind to the antibody–ligand complex, which had been previously incubated and bound.
4. After incubation, the reaction wells of the Elisa working plate were washed three times consecutively with washing solution to remove unbound antibodies. For this process, the washing solution in the kit with a concentration of 100× was diluted one hundred times with distilled water. For each wash cycle, 350 microliters of this solution prepared at a concentration of 1x was added to all wells, shaken for 60 s to allow unbound antibodies to pass into the wash solution, and then the liquid in the well was discarded. The process was repeated three times.
5. After washing, 100 microliters of Avidin Horse Radish Peroxidase (HRP) enzyme was added to each well to label antibody–antigen–biotinylated antibody complexes in the working plate. Incubation at 37 °C for thirty minutes was performed to allow binding of this enzyme marker molecule, followed by five consecutive washes to remove unbound enzyme components.
6. After the enzyme labeling step, 90 microliters of substrate was added to each well to react with the enzyme. The Elisa plate was incubated at 37 °C for 15 min to complete this reaction.
7. A total of 50 microliters of stop solution was added to all wells to stop the reaction. Then, the Elisa plate was placed in the Elisa plate reader (BioTek, ELx 50) and the reading stage was started.
8. Using the Gen 5 program, the absorbances of the reacted samples belonging to the standards and study samples at different concentrations in each well of the Elisa plate were read at a wavelength of 450 nanometers.
9. The concentration–absorbance graph was plotted against the absorbance values of the standards of known concentrations to obtain the standard working curve and reaction equation.

Using the standard reaction curve and equation, concentrations were calculated based on the measured absorbance of all patient and control group study samples. All results were expressed in picogram/mL and used in data analysis.

Concentrations of sNRP-1 standard used in the study:

The lyophilized standard vial included in the kit was centrifuged in a 4000× *g* centrifuge for ten minutes and then dissolved using one milliliter of diluent suitable for standard dissolution; 1000.00 picogram/mL standard solution was obtained. After waiting for twenty minutes for complete dissolution, the solution was homogenized using a vortex. Then, using the standard diluent, standard solutions of different concentrations were obtained by serial dilution method from 1000.00 pg/mL standard.

Prepared sNRP-1 standard concentrations were as follows:

- 1000.00 pg/mL
- 500.00 pg/mL
- 250.00 pg/mL
- 125.00 pg/mL
- 62.50 pg/mL
- 31.25 pg/mL
- 15.62 pg/mL
- 0 pg/mL (standard diluent)

### Statistical Analysis

Data were entered into the Statistical Package for the Social Sciences (IBM^®^ SPSS Statistics for Windows, Version 23.0, Armonk, NY, USA). The normality of distributions was assessed using the Kolmogorov–Smirnov test. For quantitative variables, normal distributions were expressed as mean and standard deviation (SD), while non-normal distributions were expressed as median and interquartile range (IQR). The Student’s t-test was used to compare normally distributed continuous variables. For non-normally distributed variables, the Mann–Whitney U test was used. Pearson’s chi-square test was applied for comparisons of categorical variables. If the sample size was small (≤5), Fisher’s exact test was used. A *p*-value of <0.05 was considered statistically significant.

Binary logistic regression was used to identify variables that could predict PE. Pregnant women in the control group served as the reference category. In univariate analyses, variables with a *p*-value < 0.05 were included in the multivariate analysis. The enter method was applied for variable inclusion in this analysis. Receiver operating characteristic (ROC) curves were generated, and the areas under the curves (AUCs) were calculated to assess the predictive value of maternal plasma NRP-1 and fetal cord plasma NRP-1 for PE. Additionally, the 95% confidence intervals (CIs) for each AUC were determined. In this study, an AUC value of ≤0.599 was generally considered to indicate a lack of predictive ability.

The “optimal” cut-off points for maternal plasma NRP-1 and fetal cord plasma NRP-1 were determined by identifying the values that provided the best sensitivity and specificity, calculated using ROC analysis. The negative predictive value (NPV) and positive predictive value (PPV) for these cut-off points were also calculated. Patients were divided into two groups based on these thresholds: those with values above and below the cut-off points. The PE rate was calculated for each group, and the odds ratio (OR) with a 95% confidence interval (CI) was calculated for those with high values compared to those with low values.

The correlation between maternal plasma NRP-1 and fetal cord plasma NRP-1, and factors such as age, body mass index (BMI), gestational age, gestational week, newborn weight, and hemoglobin levels, was analyzed using Spearman correlation analysis. The correlation coefficient (rho) was calculated, and interpreted as follows: a rho value < 0.2 indicated a very weak correlation, 0.2–0.4 indicated a weak correlation, 0.4–0.6 indicated a moderate correlation, and >0.6 indicated a high correlation. A negative correlation coefficient indicated an inverse relationship (e.g., one variable increases while the other decreases), while a positive coefficient indicated a direct relationship (e.g., both variables increase or decrease together). A *p*-value of <0.05 was considered statistically significant throughout the study.

## 3. Results

There were 28 pregnant women in the PE group and 25 pregnant women in the normotensive group. No significant differences were observed between the two groups in terms of age, BMI, gravida, parity, history of surgery, or previous cesarean section (*p*: 0.58, 0.15, 0.58, 0.49, 0.41, 0.74, respectively). However, there were statistically significant differences between the groups regarding gestational week, presence of comorbidities, hypertension (HT), diabetes mellitus (DM), history of PE, and protein levels in spot urine. Comorbidities, HT, and DM were significantly more prevalent in the PE group compared to the normotensive group (*p* < 0.001, 0.002, 0.007, respectively). None of the pregnant women in the normotensive group had a history of PE or protein positivity in spot urine, while 52.6% of women in the PE group had a history of PE and 89.3% showed protein positivity in spot urine. These differences were statistically significant (*p* < 0.001 and *p* < 0.001, respectively). A comparison of the demographic and clinical data of the two groups is shown in Table 1.

A comparison between pregnant women with PE and normotensive pregnant women regarding the mode of delivery and neonatal outcomes is shown in Table 2. Statistically significant differences were found between the groups in terms of 1st and 5th minute APGAR scores and the need for NICU admission. In the PE group, APGAR scores were lower, and the NICU admission rate was higher.

There were no significant differences between the two groups in terms of newborn sex, newborn length, and newborn weight (*p*: 0.35, 0.28, 0.75, respectively). However, significant differences were observed in mode of delivery, 1st and 5th minute APGAR scores, and NICU admission rates (*p*: 0.01, 0.002, <0.001, 0.004, respectively). Pregnant women in the PE group had a significantly higher rate of cesarean deliveries compared to the control group. Both 1st and 5th minute APGAR scores were significantly lower in the newborns of pregnant women with PE compared to those in the control group. The NICU admission rate was 8% for newborns of normotensive pregnant women and 42.9% for newborns of pregnant women with PE, with this difference being statistically significant.

The comparison between pregnant women with PE and normotensive pregnant women in terms of CBC parameters, maternal plasma NRP-1, and fetal cord plasma NRP-1 is shown in Table 3. A statistically significant difference was found in maternal plasma NRP-1 levels between the two groups (*p* < 0.05). However, no significant differences were observed between the groups in terms of hemoglobin, platelets, AST, ALT, and fetal cord plasma NRP-1 (*p*: 0.73, 0.83, 0.56, 0.73, respectively). Maternal plasma NRP-1 was significantly lower in the normotensive group compared to the PE group (median 473.3 pg/mL vs. 587.7 pg/mL, respectively).

**Figure 1:**
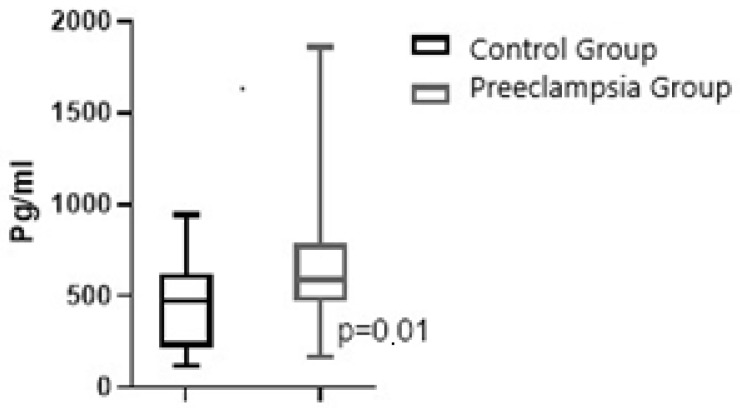
Graphical representation of the comparison between groups in terms of maternal plasma NRP-1, showing the median along with the minimum and maximum values. · *p* = 0.01.

**Figure 2:**
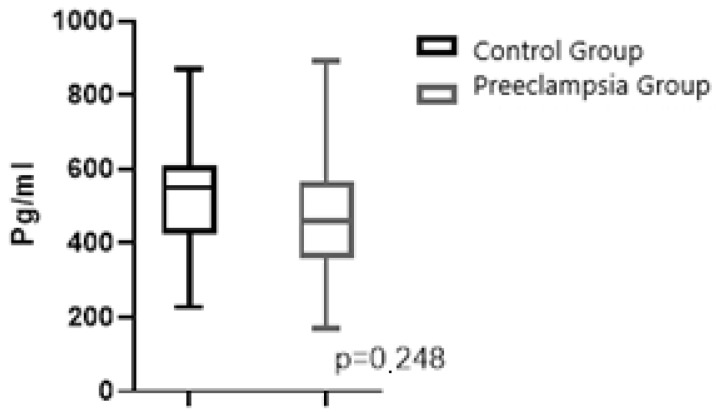
Graphical representation of the comparison of groups in terms of maternal plasma NRP-1 (median with minimum, maximum values).

Figure 1 and Figure 2 present graphical representations comparing maternal plasma NRP-1 and fetal cord plasma NRP-1 (including minimum, maximum values, and median) between pregnant women with PE and normotensive pregnant women.

The results of the binary logistic regression analysis, conducted to identify variables that may predict PE, are shown in Table 4. The presence of HT and maternal NRP-1 levels were found to be associated with the predictability of PE (*p* < 0.05). Although the rate of PE history was statistically higher in the PE group compared to the control group, this variable was excluded from the multivariate analysis as there were no cases of PE history in the normotensive group. Similarly, although gestational week and spot urine protein status were statistically different between the two groups, these factors were not included in the analysis since they are consequences, not causes, of PE. According to the multiple regression analysis, the independent risk factors for PE were the presence of HT (OR: 9.34, 95% CI: 1.02–85.48, *p*: 0.04) and maternal plasma NRP-1 levels (OR: 1.003 per unit, 95% CI: 1.000–1.005, *p*: 0.04).

The results of the ROC analysis, which examined the predictive value of maternal and fetal cord plasma NRP-1 for PE, are presented in Table 5. Our findings indicate that the best predictor was maternal plasma NRP-1 (AUC: 0.679). Based on the calculated AUC values, both maternal and fetal cord plasma NRP-1 were found to have a moderate predictive value for PE.

**Figure 3:**
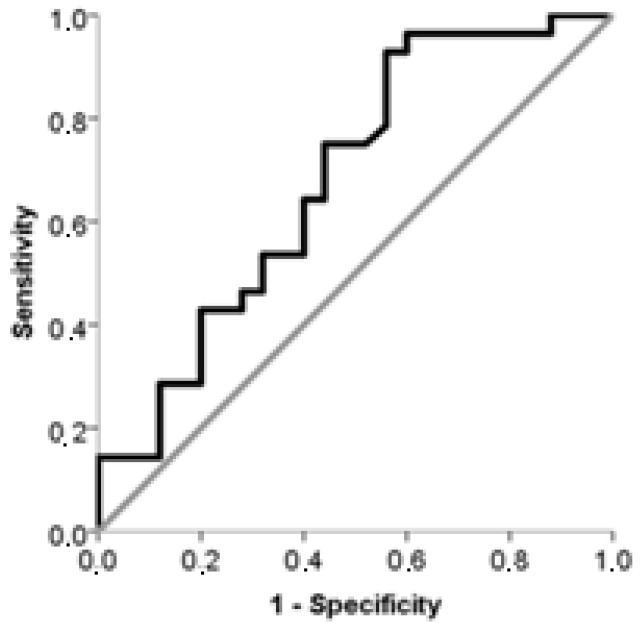
Curve of ROC analysis to examine the value of maternal plasma NRP-1 with respect to PE.

**Figure 4:**
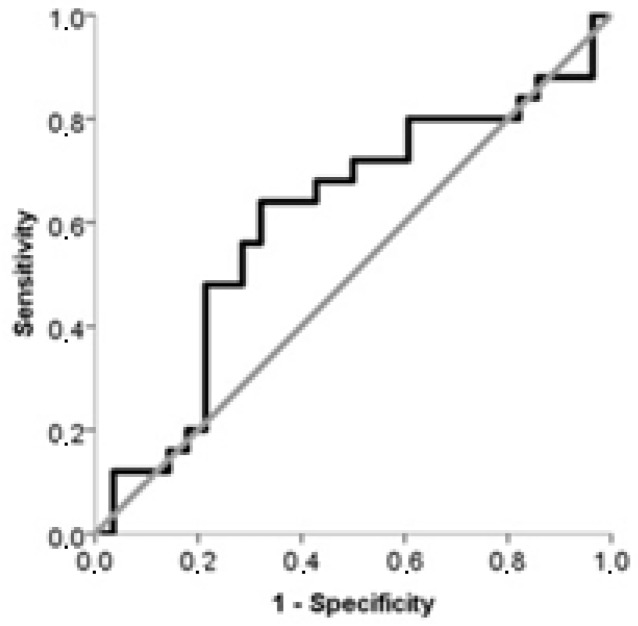
Curve of ROC analysis to examine the value of fetal cord plasma NRP-1 with respect to PE.

ROC curves of maternal plasma NRP-1 and fetal cord plasma NRP-1 for prediction of PE are shown in Figure 3 and Figure 4.

The threshold value for maternal plasma NRP-1 with the highest sensitivity and specificity for PE was determined to be 358.4 pg/mL. When pregnant women with maternal plasma NRP-1 levels above this threshold (n:40) were compared to those below it (n:13), PE was found to be significantly more likely in the higher group compared to the lower group (65% vs. 15.3%, OR: 10.21, 95% CI: 1.98–52.69, *p*: 0.002) (Table 6). Similarly, the threshold value for fetal cord plasma NRP-1 with the best sensitivity and specificity for PE was calculated as 486.5 pg/mL. When pregnant women with fetal cord plasma NRP-1 levels below this threshold (n:28) were compared to those above it (n:25), PE developed significantly less in the higher group than in the lower group (36% vs. 67.8%, OR: 3.75, 95% CI: 1.20–11.71, *p*: 0.02) (Table 6).

The correlation between maternal plasma NRP-1 and fetal cord plasma NRP-1, as well as the relationship of these variables with certain demographic and clinical characteristics of the pregnant women, are presented in Table 7. A statistically significant association was found between BMI and maternal plasma NRP-1 (*p*: 0.02), although the correlation was weakly positive (Rho: 0.304). No strong or statistically significant correlations were observed between maternal plasma NRP-1 and other variables.

## 4. Discussion

PE is a systemic condition characterized by severe vasospasm and vascular endothelial dysfunction, which typically occurs after the 20th week of gestation and may persist up to the fourth postpartum week. Due to its multifactorial pathogenesis, much of which remains unknown, early diagnosis and prevention of PE remain challenging [17]. The pathogenesis of PE is thought to involve placental dysfunction, including inadequate extravillous trophoblast invasion and insufficient remodeling of the myometrial spiral arteries [18,19]. In the maternal circulation, the balance between pro-angiogenic and anti-angiogenic proteins is disrupted by a hypoxic–ischemic environment, which contributes to endothelial dysfunction [20]. This study was designed to compare maternal plasma NRP-1 and fetal cord blood plasma NRP-1 levels in pregnancies complicated by PE and those in normotensive pregnant women.

Cord blood NRP-1 levels were also examined to address placental factors only. When maternal NRP-1 and cord blood NRP-1 levels were examined, we can say that this maternal blood value is due to placental factors based on the fact that the cord blood levels were not statistically significant but the maternal blood values were significant.

The pathogenesis of preeclampsia includes incompatibility of the placenta due to insufficient extravillous trophoblast infestation and inadequate remodeling of the myometrial spiral artery [8,9]. In the maternal circulation, the balance between pro-angiogenic and anti-angiogenic proteins is disturbed due to the hypoxic–ischemic environment. The disruption of this balance makes it susceptible to endothelial dysfunction [20].

Recent evidence has shown that the balance between pro- and anti-angiogenic factors plays an important role in the maintenance of early pregnancy and the development of preeclampsia by regulating angiogenesis at the embryo–maternal interface. Vascular endothelial growth factors (VEGF) are angiogenic factors released by the placenta, placental growth factor (PIGF). NRP-1, which specifically binds to VEGF165 and increases the binding of VEGF165 to VEGFR2, has been identified in vascular endothelial and tumor cells [15]. NRP-1, which is thought to play an important role in angiogenesis during pregnancy, is synthesized to a greater extent in the early periods than in the late periods.

Identifying risk factors for PE has been a significant focus of academic research. In a study by English et al., risk factors for PE were discussed, including preconception HT, chronic kidney disease, DM, and a history of early-onset PE [21]. The National Institute for Health and Care Excellence (NICE) guidelines identify a history of hypertensive diseases in previous pregnancies, maternal chronic kidney disease, autoimmune diseases, DM, and chronic hypertension as risk factors for PE [22]. Paré et al. found that chronic hypertension, pregestational DM, multiple gestation, African American race, prior PE, nulliparity, assisted reproductive techniques, and being overweight were all risk factors, with pregestational DM presenting the highest risk for developing PE. Notably, no significant risk association was found between advanced maternal age and PE in their study. In our study, we observed statistically significant differences between the PE and control groups regarding HT, DM, and previous history of PE. In the multiple regression analysis, a significant association was found between PE and HT, with an OR of 9.34 [23]. This OR is higher compared to other studies in the literature, which may be attributed to the relatively small sample size in our study.

Although we did not have endometriosis and adenomyosis patients in our patient and control group, there are studies on NRP-1 levels in endometriosis and adenomyosis due to the presence of angiogenesis in the etiopathology. Barberic et al. found that NRP-1 expression was significantly increased in estrogen-induced epithelial–mesenchymal transition (EMT) of endometrial cells in adenomyosis, particularly in the ectopic endometrium [24]. NRP-1-overexpressing endometrial tissues exhibited high levels of NRP-1 expression, characterized by a mesenchymal phenotype. This phenotype was marked by the downregulation of E-cadherin and occludin, the upregulation of α-SMA and N-cadherin, and enhanced cell migration. In a study by Barberic et al., the relationship between NRP-1 and endometriosis was examined. It was found that NRP-1 levels were elevated in cases of stage 2–4 endometriosis compared to the control group. Using 11 mg/L as the cut-off value for NRP-1 in ROC analysis, the AUC was calculated to be 0.97 [24]. With this threshold, sensitivity was 99.3%, and specificity was 97.8%, demonstrating a high level of diagnostic effectiveness.

Recent evidence has highlighted the critical role of the balance between pro-angiogenic and anti-angiogenic factors in maintaining early pregnancy and in the development of PE by regulating angiogenesis at the embryo–maternal interface. VEGF and PIGF are key angiogenic factors released by the placenta. NRP-1, which specifically binds VEGF165 and enhances its interaction with VEGFR2, has been identified in vascular endothelial and tumor cells [13,14]. NRP-1, believed to play a significant role in angiogenesis during pregnancy, is synthesized at higher levels in the early stages of pregnancy compared to the later stages. In a study by Awoyemi et al., pregnant women with normal pregnancies were compared with those diagnosed with PE [25]. The study’s findings showed that NRP-1 expression was confined to small syncytiotrophoblast membrane extracellular vesicles, but not to medium or large syncytiotrophoblast extracellular vesicles. Additionally, NRP-1 was co-expressed with placental alkaline phosphatase. They concluded that NRP-1 was present in small syncytiotrophoblast extracellular vesicles but absent in medium/large vesicles in placentas from both PE and healthy pregnancies. Arad et al. compared the placental tissue of 20 normotensive pregnant women and 19 pregnant women with severe PE after performing immunohistochemical staining with an anti-human NRP-1 antibody [26]. They found that NRP-1 expression in syncytiotrophoblasts was lower in the placentas of women with PE [26]. To date, there has been no study directly comparing maternal plasma NRP-1 and fetal cord plasma NRP-1 levels in normotensive pregnant women and those with PE. Cackowskı et al. compared NRP-1 and VEGF levels in placental samples from 16 normotensive and PE animal models using reverse transcriptase–polymerase chain reaction (RT-PCR) and western blot analysis [27]. They observed that NRP-1 and VEGF expression was lower in the placentas of guinea pigs with PE [27].

Studies on tissue NRP-1 expression on placental pathology have focused on decreased expression of NRP-1 in the syncytiotrophoblast villous layer in preeclampsia [28]. However, in our study, sNRP-1 levels were examined in maternal blood and cord blood. sNRP-1 is an anti-angiogenic molecule that binds to VEGF165 and PIGF [29,30]. Overexpression of sNRP-1 in tumor cells leads to damage of the vasculature and causes apoptosis of the tumor cell [31]. In a study, sNRP-1 was shown to inhibit the upregulation of inflammation and edema caused by VEGF overexpression in cutaneous delayed-type hypersensitivity reactions [32]. Another study showed that sNRP-1 plays a role in the inhibition of human breast carcinoma cell migration. This revealed the antagonistic role of sNRP-1 in angiogenesis and tumorigenesis compared to full-length NRP-1 [28]. NRP-1 also functions as a receptor for VEGF-A and semaphorin A (SEMA 3A). SEMA 3A shows anti-angiogenic effects such as impaired endothelial cell adhesion and migration [33]. Given that VEGF165 and SEMA 3A are competitive inhibitors, their imbalance affects the extent of tumor angiogenesis and alters sNRP-1 levels [34].

When reviewing the literature, studies on intrauterine growth restriction (IUGR) groups, which have a pathophysiology similar to that of PE, are noteworthy. Maulik et al. examined the postpartum placentas of fourteen pregnant women (seven with IUGR and seven healthy pregnancies) and observed that NRP-1 release was downregulated in the IUGR group [34]. Porter et al., who measured maternal plasma NRP-1 using the ELISA method in IUGR pregnancies compared to healthy pregnancies, found a significant decrease in maternal plasma soluble NRP-1 (sNRP1) concentrations in growth-restricted pregnancies with fetoplacental circulatory disorders. However, in the same study, there was no significant difference in maternal plasma NRP-1 concentrations between the control group and IUGR pregnancies with normal umbilical artery Doppler findings [35]. Yang et al. demonstrated that increased trophoblast proliferation was associated with higher NRP-1 expression in both in vitro and in vivo models, and that this increase was regulated by the QKI-5. Additionally, QKI-5 and NRP-1 expressions were found to be decreased in placentas and trophoblasts under hypoxic conditions in PE [16]. Based on these findings, NRP-1 is thought to play a role in the development and exacerbation of PE. In our study, an elevated NRP-1 level was found to be a risk factor for PE. The OR for each pg/mL increase in maternal serum NRP-1 level was 1.003. Furthermore, the AUC for values above the maternal serum NRP-1 cut-off value of 358.4 pg/mL was 0.679, with a sensitivity of 92.8%.

## 5. Conclusions

In order to clearly evaluate the relationship between NRP-1 and PE, future studies involving larger cohorts of pregnant women from diverse ethnic and racial backgrounds across various hospitals are needed. Based on the results of our study, the measurement of maternal NRP-1 concentration shows promise as a diagnostic tool for PE. We hope that in the future, the NRP-1 biomarker will be used as a mechanistic insight.

## Figures and Tables

**Table 1:** Comparison of demographic and clinical data of the groups.

Variables	Normotensive Group (*n*:25)	PE Group (*n*:28)	*p* Value
**Age, year, mean / SD**	28.5/6.8	29.6/7.0	0.58
**BMI, kg/m^2^ median/IQR**	27.1/5.2	29.8/6.9	0.15
**Gestational week, week, median/IQR**	38/2	37/2	**0.007**
**Gravida, n, median/IQR**	2/2	2/2	0.58
**Parity, n, median/IQR**	1/2	1/2	0.49
**Presence of comorbidities, n/%**	2/8	17/60.7	**<0.001**
**Presence of HT, n/%**	1/4	11/39.3	**0.002**
**Presence of DM, n/%**	1/4	7/25	**0.007**
**History of operation, n/%**	8/32	12/42.9	0.41
History of sectio, n/% *	8/42.1	9/47.4	0.74
History of preeclampsia, n/% *	--	10/52.6	**<0.001**
**Protein in TIT, n/%**			**<0.001**
**Negative**	25/100.0	3/10.7	
**Positive**	--	25/89.3	

Bold *p* values indicate statistical significance. * n: number; SD: standard deviation; IQR: interquartile range; BMI: Body Mass Indeks; HT: Hypertension; DM: Diabetes Mellitus.

**Table 2:** Comparison between groups in terms of mode of delivery and neonatal outcomes.

Variables	Control Group (*n*:25)	Preeclampsia Group (*n*:28)	*p* Value
**Mode of delivery, n/%**			**0.01**
**Normal**	16/64.0	8/28.6	
**Sectio**	9/36.0	20/71.4	
**Baby’s gender, n/%**			0.35
**Female**	13/52.0	11/39.3	
**Male**	12/48.0	17/60.7	
**Baby’s height, cm, median/IQR**	51.0/3.0	50.0/4.5	0.28
**Baby’s weight, g, median/IQR**	3220.0/630.0	3177.5/760.0	0.75
**APGAR 1st min, median/IQR**	8/2	7/2	**0.002**
**APGAR 5th min, median/IQR**	9/1	8/2	**<0.001**
**NICU need, n/%**	2/8	12/42.9	**0.004**

Bold *p* values indicate statistical significance. n: number; IQR: interquartile range; g: gram; cm: centimeter; APGAR: activity, pulse, grimace, appearance, respiration; Min: minute; NICU: neonatal intensive care unit.

**Table 3:** Comparison of hemogram parameters and maternal plasma NRP-1 and fetal cord plasma NRP-1 between groups.

Variables	Control Group (n:25)	Preeclampsia Group (n:28)	*p* Value
**Hb, g/dL, mean/SD**	11.5/1.4	11.6/1.5	0.73
**Platelet 10^9^/L, median/IQR**	250.0/106.5	228.5/83.3	0.83
**AST, U/L, median/IQR**	21.0/8.0	18.5/19.8	0.56
**ALT, U/L, median/IQR**	11.0/6.5	9.5/6.5	0.73
**Maternal Plasma NRP-1, pg/mL, median/IQR**	473.3/402.5	587.7/313.0	**0.02**
**Fetal Cord Plasma NRP-1, pg/mL, mean/SD**	520.6/162.8	469.2/157.1	0.248

Bold *p* values indicate statistical significance. n: number; SD: standard deviation; IQR: interquartile range; AST: aspartate aminotransferase; ALT: alanine aminotransferase; NRP-1: neuropilin-1.

**Table 4:** Determination of risk factors affecting the development of PE by multivariate analysis.

Variables	Odds Ratio	95%CI	*p* Value
**Presence of HT**	9.34	1.02–85.48	**0.04**
**Presence of DM**	6.67	0.66–66.90	0.106
**Maternal NRP-1 (for each pg/mL increase)**	1.003	1.000–1.005	**0.04**

Bold *p* values indicate statistical significance.

**Table 5:** Results of ROC analysis to examine the predictive value of maternal and fetal cord plasma NRP-1 with respect to PE.

Variable	AUC	95%CI	*p*	Treshold Value (pg/mL)	Sens. (%)	Spes. (%)	NPV (%)	PPV (%)
**Maternal plasma NRP-1**	0.679	0.537–0.801	**0.01**	>358.4	92.8	44.0	84.6	65.0
**Fetal cord plasma NRP-1**	0.606	0.462–0.737	0.193	≤486.5	67.8	64.0	64.0	67.9

Bold *p* values indicate statistical significance; Sens.: Sensitivity; Spes.: Specificity; AUC: Area under curve; NPV: negative predictive value; PPV: positive predictive value; CI: confidence interval; NRP-1: neuropilin-1.

**Table 6:** Separation of patients into two groups as below and above thresholds according to the best thresholds detected and calculation of PE development rate.

Variable and Treshold Value	Groups by Treshold Value	No Preeclampsia (n:25)	Preeclampsia (n:28)	Odds Ratio	95%CI	*p* Value
**Maternal plasma NRP-1** **(>358.4 pg/mL)**	Low (n:13), n/%	11/44	2/7.1	10.21	1.980–52.696	**0.002**
	High (n:40), n/%	14/56	26/92.9			
**Fetal cord plasma NRP-1** **(≤486.5 pg/mL)**	Low (n:28)	9/36	19/67.9	3.75	1.202–11.716	**0.02**
	High (n:25)	16/64	9/32.1			

Bold *p* values indicate statistical significance. n: number; CI: confidence interval; NRP-1: neuropilin-1.

**Table 7:** Correlation of demographic data between maternal plasma NRP-1 and fetal cord plasma NRP-1.

*Maternal plasma* Correlation with NRP-1	Rho	*p* Value
**Fetal cord plasma NRP-1 (pg/mL)**	0.182	0.192
**Age (year)**	0.034	0.809
**BMI (kg/m^2^)**	0.304	**0.02**
**Gestational week (week)**	−0.001	0.993
**Baby’s weight (g)**	−0.215	0.123
**Hemogram (Hg)**	0.120	0.392
***Fetal cord plasma*** ***Correlation with NRP-1***	**Rho**	***p* Value**
**Age (year)**	0.103	0.464
**BMI (kg/m^2^)**	−0.029	0.839
**Gestational week (week)**	−0.031	0.823
**Baby’s weight (g)**	0.013	0.926
**Hemoglobulin (Hb)**	0.018	0.900

Bold *p* values indicate statistical significance. Rho: spearman correlation coefficient.

## Data Availability

Data are contained within the original article.
